# Long-Term Effects of Environmental Lead on Erythropoietin Production in Young Adults: A Follow-Up Study of a Prospective Cohort in Kosovo

**DOI:** 10.1155/2020/3646252

**Published:** 2020-12-28

**Authors:** Pashko R. Camaj, Joseph H. Graziano, Emine Preteni, Dusan Popovac, Nancy Loiacono, Olgica Balac, Pam Factor-Litvak

**Affiliations:** ^1^Department of Environmental Health Sciences, Mailman School of Public Health, Columbia University, New York City, NY, USA; ^2^Independent Researcher, Mitrovica, Kosovo; ^3^University of Prishtina, Prishtina, Kosovo Medical Faculty (Retired); ^4^Department of Epidemiology, Mailman School of Public Health, Columbia University, New York City, NY, USA

## Abstract

**Background and Aims:**

Epidemiologic cross-sectional studies examining the relationship between environmental lead (Pb) exposure and erythropoietin (EPO) production have reported contrasting results. It is unknown, however, if exposure to Pb earlier in life has an effect on EPO production later in life. Here, using a prospective study, we evaluate the association between prenatal, early childhood, and concurrent Pb exposure and EPO concentration in young adulthood.

**Methods:**

From our prospective birth cohort study in Mitrovica (a mining town) and Pristina (a control town), Kosovo, from 1985 to 1998, we located and assessed blood lead concentration (BPb) and serum EPO in 101 participants (mean age 24.9 years) in 2011. We examined the association between BPb and EPO, stratified by hemoglobin (Hgb), and controlling for potential confounders.

**Results:**

These results resemble the findings in the original full cohort at 4.5 and 6.5 years of age, at which time we reported that the maintenance of a normal Hgb required increased EPO production among participants exposed to high levels of environmental Pb. In contrast, when the original cohort was 9.5 and 12 years of age, they were no longer capable of hyper-production of EPO in order to maintain normal levels of Hgb, suggestive of cumulative toxicity to the peritubular cells of the kidney that are responsible for EPO synthesis.

**Conclusion:**

Our results, along with previously reported findings on this cohort, suggest that a dramatic reduction of Pb exposure may allow for a reversal of the impact that prolonged Pb exposure may have on EPO production.

## 1. Introduction

Environmental lead (Pb) exposure has long been known to be associated with anemia [1–3] likely due to a range of mechanisms including shortened erythrocyte survival [4, 5], ineffective erythropoiesis [6], and inhibition of enzymes of the heme synthetic pathway [7–9]. Evidence of subclinical effects of Pb exposure on hematopoiesis is also reported in individuals with normal levels of hemoglobin. For example, Grandjean et al. [10] reported that, following the donation of a unit of blood, Pb-workers demonstrated “delayed blood regeneration” in comparison to non-worker controls. That report led Graziano et al. [11] to hypothesize that the production of erythropoietin (EPO), the renal hormone that regulates red cell production [12], may be inhibited by Pb exposure. Approximately eighty percent of EPO is produced in the kidneys in a feedback process as a reaction to reduced oxygen delivery to the tissues, and the remaining twenty percent is produced in the liver [12, 13]. In the kidney, EPO is primarily produced in the proximal renal tubules [13–15] where Pb is known to accumulate. Graziano et al. found that serum EPO levels declined as BPb increased in a population of chronically exposed pregnant women with a wide range of blood Pb (BPb) and Hgb levels, compared to pregnant women without a point source of lead exposure [11]. The latter study took place in two towns in Kosovo (then Yugoslavia): Mitrovica, the site of Pb mining and smelting operations, and Prishtina, relatively unexposed city 25 miles away.

We subsequently described pregnancy outcome among 1,502 women in those two towns [16, 17] and went on to study childhood development through age 12 years in a subset of their offspring [18–20]. In addition, we examined the associations between BPb and serum EPO in the children at ages 4.5, 6.5, and 9.5 years. We reported that, at age 4.5, children required *hyperproduction* of EPO to maintain normal Hgb levels, but with increasing age (and exposure from the smelter operations) this compensatory mechanism gradually failed, perhaps indicative of cumulative effects of Pb on renal endocrine function [21].

In the current study, we re-examine a subset of this pediatric cohort, at age 25 years, to test the hypothesis that—as in their mothers—chronic environmental Pb exposure would lead to an inability to mount an appropriate compensatory increase in EPO when Hgb is relatively low. Between the time of the last childhood assessment, in 1998, and the present study, Pb exposure (and BPb levels) declined dramatically as a result of the shutdown of the lead smelting operations in Mitrovica.

## 2. Methods

### 2.1. Study Design

The original cohort has been described in previous publications [16, 17, 22, 23]. Briefly, pregnant women were recruited between 1984 and 1985 in two towns, Mitrovica, the site of the Trepça mines, smelter, and battery plant, and Prishtina, the capitol, which was relatively unexposed. Offspring were followed every 6 to 12 months for BPb, hemoglobin (Hgb), erythrocyte protoporphyrin (EP), and serum ferritin (SF) measures, neurocognitive development, and physical examinations until age 12.5. We established a biorepository for future analyses, such as the one reported here. In 2011, we located and identified 101 members of the original cohort and requested their participation in a follow-up study, in which each participant would be evaluated once to assess their BPb, serum EPO, and Hgb levels. Follow-up was carried out in a central location in each town. Questionnaires were administered in the participant's primary language, Albanian or Serbian. A trained laboratory technologist and a physician carried out the fieldwork. All blood samples were refrigerated immediately at 4° Celsius and then frozen at −20°C prior to shipment to Columbia University for analysis. While we contacted and recruited study participants remaining in both towns, we encountered more challenges finding and recruiting the participants from Prishtina. This may be since the city expanded greatly in the past 8–10 years corresponding to changes in the ethnic makeup of the population. In addition, once contacted, those in Prishtina were less interested, and more of them refused, likely due to lack of perception of the beneficial aspects of the study. Another factor that may have influenced the recruitment may have been that most of the pre-war Serbian population in Prishtina had been uprooted from Kosovo at the beginning of NATO campaign in 1999. However, the Serbian population in Mitrovica (northern part of the city) for the most part has remained in place. There may have been some temporary migration during the few months at the height of the war, but it has been reported that most had returned to their homes shortly after. Similarly, the Albanian population in Mitrovica has remained very much intact with a similar pattern of temporary migration during the few months (1–3 months) of the height of the hostilities. Their temporary migration was mostly to camps in neighboring Albania and Montenegro. However, as the hostilities diminished, the overwhelming majority returned to their homes.

### 2.2. Data Collection and Laboratory Analyses

Demographic information and other cohort characteristics were collected via questionnaire. In addition, we accessed existing data from mid-pregnancy to ascertain the life-style characteristics of the then-pregnant mothers of cohort members.

(i) Blood samples were collected in EDTA vacutainers by venipuncture from each participant. BPb levels were analyzed using a Graphite Furnace Atomic Absorption Spectrophotometer (GFAAS), using a method modified from Fernandez and Hilligoss [24]. Hgb concentrations were measured using the standard cyanmethaemoglobin method. An enzyme linked immunosorbent assay (ELISA) kit was used for the quantitative determination of serum-EPO concentrations (Quantikine IVD Human Erythropoietin). Quality Control (QC) samples were used, at the beginning of each run, after every 10^th^ sample, and at the end of the run. Columbia University's laboratory participates in the Center for Disease Control and Prevention quality control program for BPb; during the study, the interclass correlation between the expected and observed BPb was 0.99. Interprecision coefficients of variation for serum-EPO were 2.5% for quality control samples and 2.8% for study samples. BPb measurements from childhood were already available from the parent cohort study, measured every six months from birth through age 12 years; those measurements were conducted in the same laboratory using the same method.

### 2.3. Statistical Analyses

Descriptive analyses compared the distributions of continuous variables using *t*-tests and categorical variables using chi-square tests between towns. We used unadjusted linear regression models to estimate the crude associations between current BPb levels and EPO. Our main hypothesis, to examine associations between concurrent BPb and EPO, was tested using linear regression models controlling for concurrent Hgb concentration, the most important predictor of EPO [19], ethnicity, sex, body mass index (BMI), smoking, employment status, and educational attainment. Potential confounders were retained in the final models if the estimated coefficient relating BPb to EPO changed at least 10% with their inclusion. Ethnicity, age, education and employment status were omitted from the final models since they did not meet the criteria for potential confounding. BPb measures were available for each subject from birth through 12.5 years of age, and at the follow-up. We first examined the associations with BPb measured at the time of follow-up. Second, we calculated cumulative lead exposure, using the trapezoidal area under the curve for various age periods: from birth through age 2, ages 2–4, 4–7, and ages 7–12. Third, we examined the associations between EPO and the total area under the BPb vs. age curve. The distribution of BPb was not normally distributed so we utilized a base 10 logarithmic transformation. All analyses were performed using SAS version 9.3 (SAS Institute, Carry, NC).

## 3. Results

The original cohort consisted of 574 children; the sample size was reduced to 178 at the follow-up at age 12.5 years, likely due to the wartime situation. From the original 574 children cohort, we assembled 101 participants in 2011 when they were approximately 25 years old. Over 80% of the follow-up sample were from the exposed town, Mitrovica, compared to 55% in the original birth cohort. Those residing in Prishtina were better educated and more likely to be employed compared to those in Mitrovica. Smoking was more prevalent in Prishtina (66% vs. 29% in Mitrovica). The sex distribution reflected that of the original cohort. Biomarkers and anthropometric characteristics are also described in Table 1. In addition, Supplemental Table 1 compares characteristics of the follow-up sample to the original cohort. The current Mitrovica cohort (exposed town) more closely resembles the original cohort than that of Prishtina (non-exposed town). This is likely due to a possible bias in the sample of the study participants in Prishtina where the sample size was also significantly smaller compared to that of Mitrovica (shown in Supplemental Table 1).

**Figure 1:**
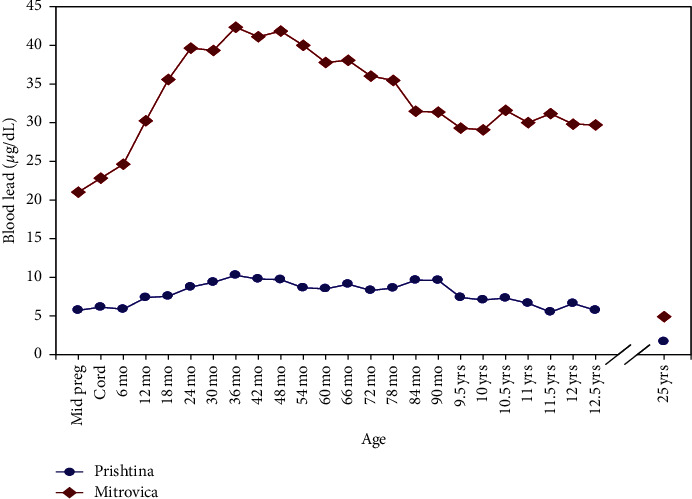
Average BPb levels in Mitrovica (top) and Prishtina (bottom) for the first 12.5 years of their lives and at 25 years of age (*N* = 101).

Mean BPb concentrations remained significantly higher in Mitrovica residents compared to those from Prishtina, even though the lead smelting plant drastically diminished its operations in late 1990's and closed at the onset of the war in 1999. Smelting operations have remained closed and only ore mining operations have resumed since 1999, albeit at a reduced capacity. Average BPb concentrations for these 101 participants from birth through age 12.5 and at age 25 are illustrated in Figure 1. Concurrent BPb levels ranged from 1.41–16.4 *μ*g/dl and 0.69–3.51 *μ*g/dl in Mitrovica and Prishtina, respectively. At age 12.5 years, the mean BPb concentrations were 30.6 *μ*g/dl (SD = 8.8 *μ*g/dl) in Mitrovica and 6.1 *μ*g/dl (SD = 1.6 *μ*g/dl) in Prishtina. Peak BPb occurred at 36 months of age in both towns, with peak values of 42.3 *μ*g/dl in Mitrovica and 10.3 *μ*g/dl in Prishtina. Mean Hgb was 0.39 g/dl lower in Mitrovica than Prishtina, but this difference was not significant. However, the mean serum EPO concentration of 10.99 mIU/ml in Mitrovica was significantly higher than that of 8.49 mIU/ml in Prishtina (*P* < 0.04). Overall, 17% of participants had Hgb < 11.0 g/dL and ∼32% had Hgb < 13 g/dL.

In Table 2, we present the mean values of BPb, Hgb, and EPO of the current sample as compared to those of all children in the cohort at birth and at ages 2, 7, and 12 years. With one exception, the mean values of BPb, Hgb, and EPO of the current sub-sample did not differ from the original study sample; the exception is that, at age 2, the current study sample had lower Hgb levels than the overall sample (10.64 vs. 11.13 g/dl, respectively; *p*=0.017), a finding likely due to chance as there were many comparisons made in this table.

**Figure 2:**
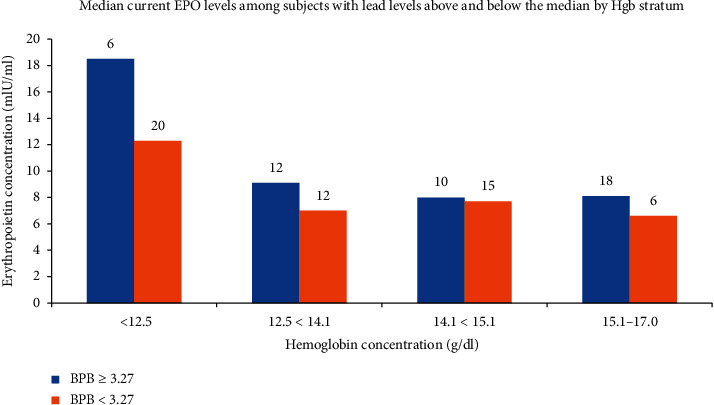
Mean EPO concentrations stratified by Hgb concentrations. Numbers above bars indicate the number of participants each bar represents.

**Figure 3:**
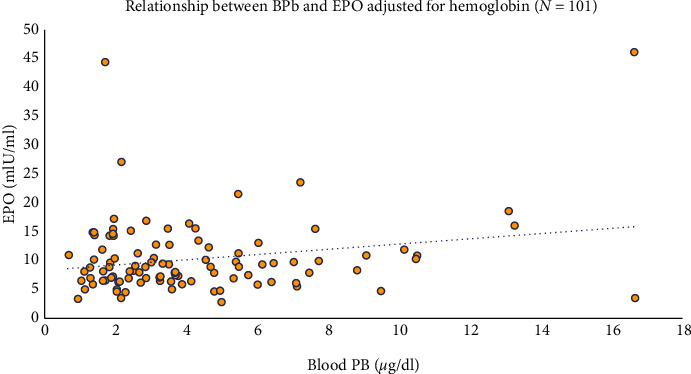
Relationship between BPb and EPO adjusted for hemoglobin.

**Figure 4:**
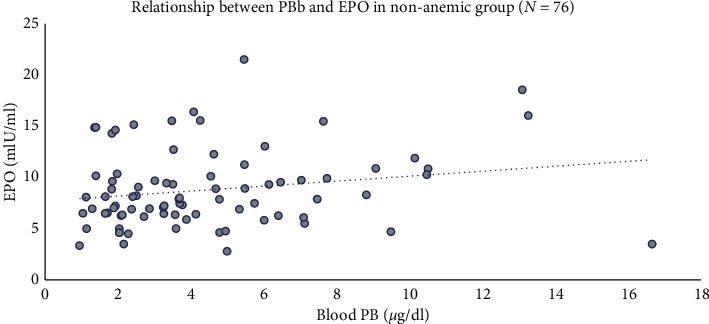
Relationship between BPb and EPO in nonanemic subjects (Hgb ≥ 12.5 g/dl).

**Figure 5:**
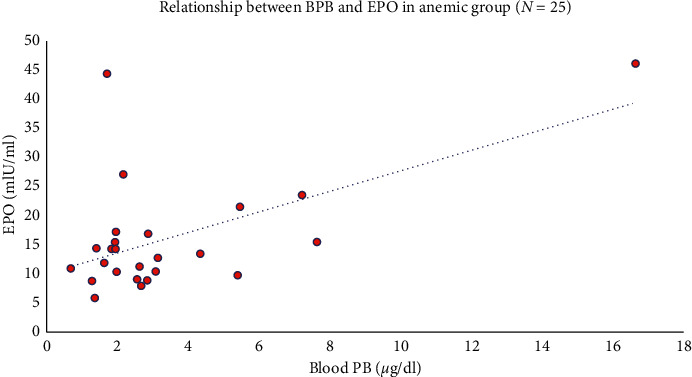
Relationship between BPb and EPO in anemic subjects (Hgb < 12.5 g/dl).

Hgb concentration is the strongest predictor of serum EPO levels. We therefore first examined the relationship between BPb and serum EPO by stratifying the sample into quartiles of Hgb (Figure 2) and stratifying by those above and those below the median BPb concentration of 3.27 *μ*g/dl. As shown in the figure, within each Hgb quartile, those with lower BPb values tended to have higher serum EPO concentrations, most notably in the lowest quartile of Hgb, where the physiologic demand for compensatory EPO synthesis is most pronounced. A simple linear regression model relating log BPb to log serum EPO did not find a significant relationship between the two variables (estimated *β* = 0.05; 95% CI −0.09, 0.20) (Table 2). However, when adjusted for Hgb as a continuous variable, shown in Table 3, the relationship became significant (estimated *β* = 0.19; 95% CI 0.06, 0.31). This finding is illustrated graphically in Figure 3. The relationships between log BPb and log EPO dichotomized by Hgb (less than or greater than or equal to 12 g/dl) are illustrated graphically in Figures 4 and 5. In the multivariable analysis, significant positive associations were found between cumulative BPb (AUC between birth and age 2 years, AUC between ages 2 and 4 years, and AUC between ages 4 and 7 years) and EPO; however, the estimated associations diminished with child age, after adjustment for Hgb. Although no associations were found between cumulative BPb between ages 7 and 12 and EPO, the sign of the estimated association changed from hyperproduction of EPO to hypoproduction of EPO (Supplemental Table 2).

## 4. Discussion

The participants in the current study had last been evaluated in 1998 at age 12.5 years. By 2011, at age 25, the mean BPb had decreased from 29.7 to 4.91 *μ*g/dL in Mitrovica, and from 5.73 to 1.67 *μ*g/dL in Prishtina. By that time, the operations at “Trepça Mines and Smelters” in Mitrovica had been completely shut down for more than a decade, undoubtedly leading to a reduction of airborne Pb and less ongoing exposure. Nevertheless, the legacy of early childhood Pb exposure was still apparent at the age of 25, as we found higher mean BPb of 4.91 *μ*g/dL, and range of 1.41–16.4 *μ*g/dL, in that town.

In this study, we examined the relationship between environmental Pb exposure and serum EPO in a group of young adults in whom this relationship has been followed longitudinally over time. We tested the hypothesis that higher levels of prenatal and early life Pb exposure are associated with decreased EPO production later in life. Lead exposure was estimated using past measurements of BPb from birth through age of 12, which were already available, as well as concurrent BPb at 25 years of age. The results show a positive association between concurrent BPb and serum-EPO levels with a significantly more pronounced slope in the regression line for the low-Hgb level participants. These results are similar to our previously reported findings in the original full cohort at 4.5 and 6.5 years of age, at which time we reported that the maintenance of a normal Hgb required hyper-production of EPO to do so, presumably a physiologic adaptation to shortened red cell survival in the face of high BPb in the Mitrovica participants [25]. In contrast, when the original cohort was 9.5 and 12 years of age, they were no longer capable of doing so, suggestive of cumulative toxicity to the peritubular cells of the kidney that are responsible for EPO synthesis [25]. In addition, the current EPO findings also contrast those reported in the anemic mothers of this study cohort [11] during pregnancy, where serum EPO levels were lower in those with higher BPb levels; those women, however, had high concurrent BPb levels, ranging from 23.1 to 36.2 *μ*g/dL.

The current findings indicate that there is a positive association between concurrent BPb and serum EPO, a finding that is particularly apparent in those with relatively lower Hgb concentrations (Figures 1 and 4). We interpret these findings to suggest that the dramatic decline in BPb concentrations that occurred during the many years since this cohort was last evaluated has allowed some recovery of renal peritubular cell function. Despite the relatively low concurrent BPb at age 25, the observed positive association between BPb and serum EPO suggests that perhaps Pb-induced effects on circulating red cells lead to shortened red cell survival, thereby creating a demand for endogenous EPO synthesis as a compensatory mechanism. Blood lead levels reflect recent exposure to Pb; however, they are also influenced by long-term exposures through efflux of lead from bone stores [26]. Even though environmental exposure to Pb may have been significantly reduced in the past 15–20 years, because of the profound difference in half-lives of Pb in various compartments (i.e., blood, tissue, and bone), constant levels of circulating BPb can be primarily attributed to a continued exchange of Pb between the bones and the blood and soft tissue. More importantly, lead in bone can, under certain conditions, be mobilized at an increased rate and released back into the systemic circulation in order to achieve the state of equilibrium [27–29]. Thus, the observed adverse health effects could be associated with current and cumulative exposures. Alternatively, it is conceivable that Pb has a lasting adverse effect on erythroid progenitor cells that leads to an inappropriate demand for EPO, which only partly recovers with reductions in exposure [30].

Increases in BPb have been reported to be associated with decreased serum EPO levels in Pb workers with BPb ranging from 30–92 *μ*g/dL [30, 31], as compared to controls with a mean BPb of 10 *μ*g/dL. A similar negative association has been reported in children by Liebelt and co-workers [32] who examined the relationship between BPb and EPO (controlling for Hgb) in 86 children with a range of BPb of 2–84 *μ*g/dL. In 2007, Sakata et al. found a negative association between BPb and serum EPO concentrations in subjects with normal renal function (mean BPb 6.4 *μ*g/dL in exposed and 2.4 *μ*g/dL in non-exposed group), indicating that Pb inhibits renal EPO production in a dose-dependent manner in persons with relatively low BPb concentrations. These investigators suggest that serum EPO concentration may serve as an early biomarker of undue Pb exposure [33]. In another study, the physiological relationship between BPb and serum EPO did not change (mean BPb 42.3 *μ*g/dL in exposed and 10.6 *μ*g/dL in non-exposed group) erythropoiesis and EPO did not seem to be influenced by BPb [34]. This outcome may have been obscured by not controlling for hemoglobin in the studied groups.

The *positive* association between BPb and EPO in our current study suggests that the relatively low concurrent BPb levels are not sufficient to impair EPO synthesis in the group that requires EPO production to respond to lower Hgb levels (anemic group). The factors that may influence the serum EPO levels, in addition to Hgb, are sex, age, and smoking [21, 35, 36]. Increased levels of EPO were found in smokers despite higher Hgb levels, suggesting that the condition of anemia in smokers may be masked by high Hgb levels [35, 37]. Smokers maintain a higher Hb level than nonsmokers [38]. A recent study suggests that higher EPO levels are associated with increased BMI [36]. In addition, increased levels EPO are positively associated with age [39]. However, little is known regarding other factors that may influence serum EPO concentrations such as ethnicity and socioeconomic status (SES). This study has several strengths. First, there was a wide range of BPb measured early in life, albeit less of a range at age 25, making possible the analysis of early life exposure and later outcomes. Second, although the follow-up was small, baseline characteristics of the participants were similar to those from the larger pregnancy cohort, especially for the exposed town (Mitrovica). Third, in our statistical analysis, we controlled for ethnicity, sex, smoking, BMI, employment status, and education as potential confounders of the outcome.

The study is limited in that we only followed a select 20% of the original sample; however, there is no reason to assume that the biological relationships would differ between those followed and those not followed. The differences in ethnicity, sex, smoking, BMI, and education likely do not affect the relationships and do not in a significant way affect EPO concentration. Furthermore, the studied sample is comparable to the childhood BPb levels of the larger cohort.

Another limitation is the 12 ½ year gap in the last childhood BPb measurements and a single measure of EPO. Because the EPO levels were measured only once for each participant, the robustness of the correlations may be affected by the inter-assay variability of the EPO level within any individual. Additionally, the study was an oversampling of highly exposed children. Furthermore, this study does not include a bone marrow biopsy, and as such, we cannot be 100% sure that the decrease in EPO is entirely attributable to lead. Lastly, the study does not include any liver function tests in this relatively young cohort. Therefore, results likely cannot be generalized to children and young adults with lower levels of lead exposure. Finally, our analysis is based on a select population residing in Kosovo and the results may not be generalizable to other populations.

## 5. Conclusion

We report that, in this group of young adults who were chronically exposed to Pb in early childhood, serum EPO concentrations responded appropriately as a function of Hgb concentration but also increased inappropriately as concurrent BPb increased. Thus, a perturbation of this renal/hematopoietic balance is still evident many years after the cessation of exogenous Pb exposure.

## Figures and Tables

**Table 1:** Sample characteristics ((%) or mean ± SD).

Variable	Prishtina (*N* = 21) *N* (%)	Mitrovica (*N* = 80) *N* (%)	*p* value
Mean age	24.9 + 0.49	24.8 + 0.48	0.49

Ethnicity			
% Albanian	95.2	68.7	0.01
% Serbian + other	4.8	31.2	

Sex			
% male	47.6	46.2	0.91
% female	52.4	53.8	

Education			
% high school or less	19.0	51.3	0.01
% college or more	80.9	48.7	

Employment status			
% employed	76.2	47.4	0.02
% unemployed/seeking employment	23.8	52.5	

Smoking status			
% current	66.6	29.3	0.01
% no	33.3	70.7	

Maternal education			
% high school or less	85.7	93.7	0.22
% college or more	14.3	6.2	

Mean BMI^*∗*^	23.0 + 2.3	24.0 + 4.5	0.15
Mean height (m)	1.7 + 0.09	1.7 + 0.09	0.55
Mean concurrent BPb^*∗∗*^ (*μ*g/dl)	1.7 + 0.7	4.9 + 3.26	<0.0001
Mean concurrent Hgb^*∗∗∗*^ (g/dl)	14.0 + 1.7	13.6 + 1.71	0.36
Mean concurrent EPO^ (mIU/ml)	8.49 + 3.74	10.99 + 7.12	0.04

Follow-up of participants in the Yugoslavia study of lead exposure and child development. ^*∗*^Body mass index, ^*∗∗*^blood lead, and ^*∗∗∗*^hemoglobin.

**Table 2:** Sample biomarker characteristics (*N* = 576) compared to current follow-up cohort of young adults (*N* = 101).

	Prishtina			Mitrovica		
Biomarkers	Original cohort	Current cohort (*N* = 21)		Original cohort	Current cohort (*N* = 80)	
Mean BPb (*μ*g/dl)						
BPb—umbilical cord	5.66 (3.5)^*∗*^ [255]^*∗∗*^	6.14 (3.5)	*p* 0.54	21.95 (7.9) [308]	2.8 (7.9)	*p* 0.39
BPb—age 2 years	9.31 (4.8) [170]	8.74 (5.3)	*p* 0.61	37.52 (12.3) [190]	39.6 (12.4)	*p* 0.21
BPb—age 7 years	7.56 (3.7) [100]	9.61 (7.5)	*p* 0.07	31.15 (10.5) [102]	31.4 (10.0)	*p* 0.87
BPb—age 12 years	6.31 (2.0) [61]	6.61 (3.0)	*p* 0.60	29.89 (9.7) [49]	29.8 (8.7)	*p* 0.58

Mean Hgb (*μ*g/dl)	Original cohort	Current cohort (*N* = 21)		Original cohort	Current cohort (*N* = 80)	
Hgb—cord	16.32 (2.4) [255]	15.90 (2.3)	*p* 0.44	15.89 (2.2) [308]	15.48 (2.3)	*p* 0.14
Hgb—age 2 years	10.76 (1.3) [170]	10.64 (1.6)	*p* 0.69	11.13 (1.5) [190]	10.64 (1.6)	*p* 0.02
Hgb—age 7 years	12.72 (0.8) [100]	12.84 (1.0)	*p* 0.55	12.83 (0.9) [102]	12.84 (1.0)	*p* 0.94
Hgb—age 12 years	13.05 (0.9) [60]	12.88 (1.0)	*p* 0.47	12.77 (0.8) [49]	12.88 (1.0)	*p* 0.51

Mean EPO (mlU/ml)	Original cohort	Current cohort (*N* = 21)		Original cohort	Current cohort (*N* = 80)	
EPO—age 4.5 years	5.6 (3.1) [97]	7.15 (6.3) [9]	*p* 0.20	7.8 (7.3) [114]	8.6 (7.6) [23]	*p* 0.63
EPO—age 6.5 years	8.2 (4.1) [83]	6.99 (2.4) [13]	*p* 0.30	9.7 (4.1) [95]	9.2 (3.8) [42]	*p* 0.50
EPO—age 9.5 years	8.8 (6.4) [113]	7.68 (3.7) [12]	*p* 0.55	8.8 (6.2) [121]	8.5 (3.5) [53]	*p* 0.74
EPO—age 12 years	9.2 (3.4) [98]	10.61 (6.0) [14]	*p* 0.18	9.6 (3.9) [111]	9.81 (6.1) [62]	*p* 0.78

( ), standard deviation; , number of the original cohort participants.

**Table 3:** Linear regression models relating (log) BPb to (log) serum erythropoietin concentrations (*N* = 101).

	*β*	95% CL
Unadjusted	0.05	−0.09, 0.20
Adjusted^*∗*^	0.19	0.06, 0.31
Adjusted ^		
^Anemic (Hgb < 12.5 g/dl)	0.41	0.13, 0.70
^Non-anemic (Hgb > 12.5 g/dl)	0.05	−0.10, 0.20

^*∗*^Adjusted for hemoglobin (continuous); ^adjusted for hemoglobin (dichotomous) with interaction term of lead (continuous) and hemoglobin (dichotomous).

## Data Availability

All pertinent data are in our study files and can be made available upon request.

## Abbreviations

EPO: Erythropoietin
Hgb: Hemoglobin
BPb: Blood lead
BMI: Body mass index.

## Supplementary Materials

Supplementary Materials Supplemental Table 1: sample characteristics comparing the original cohort (N-576) to the current cohort (N-101) in a follow-up of participants in the Yugoslavia study of lead exposure and child development. Supplemental Table 2: linear regression models for lead exposure at various periods of development and EPO at age 25.
