# Supplementary material for: Long-Term Effects of Environmental Lead on Erythropoietin Production in Young Adults: A Follow-Up Study of a Prospective Cohort in Kosovo
Source: J Environ Public Health. 2020 Dec 28;2020:3646252. doi: 10.1155/2020/3646252 (PMC7785392; doi:10.1155/2020/3646252)
Supplement: Supplementary Materials — Supplemental Table 1: sample characteristics comparing the original cohort (N-576) to the current cohort (N-101) in a follow-up of participants in the Yugoslavia study of lead exposure and child development. Supplemental Table 2: linear regression models for lead exposure at various periods of development and EPO at age 25. [file 3646252.f1.pdf]

Supplemental Table 1: Sample Characteristics Comparing the Original Cohort (N-576) to the Current Cohort (N-101) in a Follow up of Participants in the Yugoslavia Study of Lead Exposure and Child Development.

	**Prishtina**			**Mitrovica**		
**Variable**	**Original Cohort**	**Current Cohort (N=21)**		**Original Cohort**	**Current Cohort =80)**	
**Ethnicity**						
%Albanian	62.0	95.2	p-0.008	53.6	68.7	p-0.17
%Serbian and other	38.0	4.8	p-0.0007	46.4	31.3	p-0.08
**Sex**						
%Male	51.4	95.2	p-0.003	52.4	68.7	p-0.13
**Education***						
% High school or less	9.0 (4.1)**	19.0		8.9 (4.23)**	51.3	
% College or more		80.9			48.7	p-0.004
**Employment***						
% Employed	45.6	76.2	p-0.005	32.5	47.4	p-0.09
% Unemployed	55.4	23.8	p-0.003	7.5	52.5	p-0.07
**Smoking***						
% Smokers	28.3	66.6	p-0.0005	24.8	29.3	p-0.39
% Non-Smokers	77.7	33.3	p-0.0005	75.2	70.7	p-0.20

** Data for **education**, **employment** and **smoking** refers to mothers of the children in the original cohort*

***Number of **years of education** mothers of the children*

Supplemental Table 2: Linear Regression Models for Lead Exposure at Various Periods of Development and EPO at Age 25

	β	95% CL	P-Value
Univariate			
Period of Exposure			
0-2 years	0.00024	-0.000094, 0.00057	0.16
2-4 years	0.00028	0.000022, 0.00054	0.03
4-7 years	0.000129	-0.000068, 0.00032	0.2
7-12 years	-0.000062	-0.00023, 0.000107	0.47
Multivariable*			
Period of Exposure			
0-2 years	0.00043	0.000081, 0.00077	0.02
2-4 years	0.00036	0.00010, 0.0062	0.006
4-7 years	0.00016	-0.000035, 0.00035	0.11
7-12 years	-0.00003	-0.00020, 0.00013	0.66

**adjusted for ethnicity and gender*
